# Improving the Damping Properties of Nanocomposites by Monodispersed Hybrid POSS Nanoparticles: Preparation and Mechanisms

**DOI:** 10.3390/polym11040647

**Published:** 2019-04-09

**Authors:** Wei Wei, Yingjun Zhang, Meihua Liu, Yifan Zhang, Yuan Yin, Wojciech Stanislaw Gutowski, Pengyang Deng, Chunbai Zheng

**Affiliations:** CAS Key Laboratory of High-Performace Synthetic Rubber and its Composite Materials, Changchun Institute of Applied Chemistry, Renmin Street 5625, Changchun 130022, China; weiwei@ciac.ac.cn (W.W.); zyj13125823573@163.com (Y.Z.); yfzhang@ciac.ac.cn (Y.Z.); yuany@ciac.ac.cn (Y.Y.); voytek.gutowski@csiro.au (W.S.G.); pydeng@ciac.ac.cn (P.D.)

**Keywords:** monodisperse, nanocomposites, damping, POSS, liquefied

## Abstract

In this work, a series of heptaphenyl siloxane trisilanol/polyhedral oligomeric silsesquioxane (T_7_-POSS) modified by polyols with different molecular weights were synthesized into liquid-like nanoparticle–organic hybrid materials using the grafted-from method. All grafted POSS nanoparticles changed from solid powders to liquid at room temperature. Polyurethane (PU) nanocomposites with POSS contents ranging from 1.75 to 9.72 wt % were prepared from these liquefied polyols-terminated POSS with polyepichlorohydrin (POSS–PECH). Transmission electron microscopy (TEM), scanning electron microscopy (SEM) and energy dispersive spectroscopy (EDS) were used to characterize the morphology of the POSS–PECH/PU nanocomposites. The results showed that the polyol-terminated POSS particles overcame the nanoagglomeration effect and evenly disperse in the polymeric matrix. The damping factor (tan *δ*) of resultant nanocomposites increased from 0.90 to 1.16, while the glass transition temperature decreased from 15.8 to 9.4 °C when POSS contents increased from 0 to 9.75 wt %. The gel content, tensile strength and Fourier transform infrared (FTIR) analyses demonstrated that the molecular thermal movement ability of the polyurethane (PU) matrix increased with increasing POSS hybrid content. Therefore, the improvement of the damping properties of the composites was mainly due to the friction-related losses occurring in the interface region between the nanoparticles and the matrix.

## 1. Introduction

In recent decades, nanocomposites have been intensely investigated by academy and industry researchers in the field of new materials development. A large number of nanocomposites have been successfully developed and applied, becoming the key driving force for the rapid development of advanced materials for applications in aviation, aerospace, transportation and energy technologies [1,2,3,4,5,6,7,8].

Nanocomposites often exhibit excellent strength, modulus of elasticity and toughness due to the reinforcement effects of nanofillers. In these commodity engineering applications, traditional theories of polymer mechanics are usually effective in predicting and guiding the static elastic properties of composites.

It is well known that the viscous properties of polymer composites can be expressed by the loss function known as ’tangent delta’ (tan *δ*), which is attributed to 1) intra-molecular friction and molecular relaxation, 2) friction between the polymer chain and filler, 3) friction between adjacent filler particles, and 4) flexibility of the polymer chain. The value of tan *δ* is mainly determined by its viscoelastic behavior in the glass temperature (*T_g_*) transitional region. In this region, macromolecule chain segments tend to vibrate in phase with external vibrations. If numerous types of interactions exist between the polymer and filler, a broader tan *δ* transition region would be desirable. The higher the internal friction, the higher the tan *δ* value and the broader the transition region will be; thus, excellent energy dissipation (damping) performance of such composites will be observed [9,10,11,12,13,14]. However, increasing the strength and modulus of elasticity of nanocomposites usually leads to a decrease in damping properties. Researchers active in this field believe that this is because high modulus nanofillers (especially inorganic nanofillers) restrict the thermal movement of the chains in the polymer matrix.

Since 2005, many theoretical studies have demonstrated that fracturing (detaching) molecular chains from the surface of nanofillers can cause changes in conformational entropy. The associated mechanical energy loss is transformed into heat energy [15,16,17,18,19,20]. This implies that the larger the effective interface region between polymer chains and nanofillers, the greater the damping capacity of composites to attenuate vibrations or noise. As far as we know, no studies to date have shown that the damping properties of nanocomposites would continuously improve with the increase of nanofillers in addition to the matrix, although numerous studies have demonstrated that the uniform distribution of nanosized filler particles in the polymer matrix leads to reasonably good interfacial bonding strength [21,22,23,24,25]. This may be due to the serious nanoagglomeration effect in traditional nanocomposites, which leads to a decrease in the area of the interface region with the increase of nanofillers.

In this study, solid T_7_-POSS particles were liquefied by polyepichlorohydrin (PECH) oligomer grafted by a graft-from method. POSS–PECH/polyurethane (PU) nanocomposites, all exhibiting homogeneous morphology of monodispersed POSS nanocores in the PU matrix were then fabricated and characterized. Their damping performance is starkly contrasting that observed in traditional silicon dioxide (SiO_2_), T_7_-POSS or T_8_-POSS composites. The mobility of PU chain segments increases with the content of POSS–PECH. The observed high tan *δ* and broad transition regions were attributed to increased internal friction and other interactions between the polymer chain and surface-modified POSS filler.

## 2. Materials and Methods

### 2.1. Materials

PECH was synthesized from ethylene glycol with epichlorohydrin (ECH) used as the monomer and boron trifluoride etherate (BF_3_-etherate) as the catalyst (laboratory grade). Castor oil was purchased from Tongliao Chemical Reagent Factory (Tongliao, Neimenggu, China). Polyaryl polymethylene isocyanate (PAPI) was provided by Bayer (NCO 30.5-32.0 wt %, Leverkusen, Germany). T_7_-POSS was received from Hybrid Plastics, CA (Hattiesburg, MS, USA).

### 2.2. Synthesis of POSS–PECH

**Figure 1:**
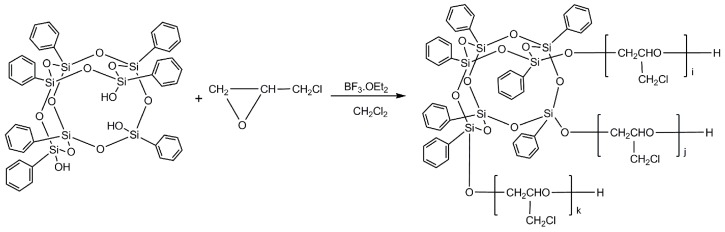
Synthetic route of POSS–PECH.

**Figure 2:**
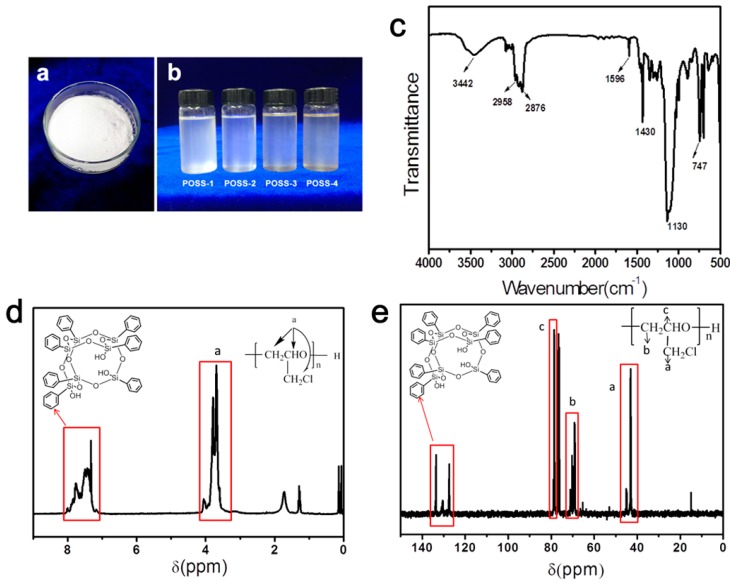
(**a**) Photograph of T_7_-POSS before liquefaction. (**b**) Photograph of T_7_-POSS after liquefaction. (**c**) FTIR spectra of POSS–PECH. (**d**) ^1^H NMR spectra of POSS–PECH. (**e**) ^13^C NMR spectra of POSS–PECH.

The synthesis route of POSS–PECH utilizing T_7_-POSS as the starting agent is shown in Figure 1. The ring-opening polymerization of epichlorohydrin was carried out in a 500 mL triple-neck flask equipped with a mechanical paddle, a temperature gauge and a dropping funnel. To the stirred solution of T_7_-POSS in dichloromethane, a precisely measured volume of boron trifluoride ether was then added as a catalyst at room temperature, and stirring was continued for 30 min. The reaction flask temperature was brought down to 18 °C using ice. Epichlorohydrin was then slowly added to the reaction mixture over a period of 4 h using a dropping funnel. The reaction mixture was then stirred for 4 h at 40 °C. The synthesized polymer solution was subsequently treated with saturated NaHCO_3_ solution and distilled water to remove unreacted diol and the initiator when the reaction was completed. The reaction mixture was then filtrated, and the solvent and small-molecule compounds were removed by vacuum distillation at 110 °C for 2 h, yielding a viscous fluid (see Figure 2b). Thus, four synthesized POSS–PECH compounds with different molecular weights were produced and are listed in Table 1.

### 2.3. Sample Preparation

POSS-based polyurethane formulations (see Table 2 for details) were prepared by a one-step method. Pre-weighted quantities of POSS–PECH, PECH and castor oil were added to a Teflon cup and then placed in the oven preheated to 85 °C for 30 min. The melt curing agent for the PUs, PAPI, was then added and mechanically stirred to achieve homogeneity. The resultant mixture was degassed under vacuum, poured into preheated glass molds coated with a mold release agent and then cured at 100 °C for 24 h. In this study, a series of polyurethane samples was prepared by changing the isocyanate index (R) and the addition of POSS–PECH.

### 2.4. Gel Extraction Experiments

Samples weighing approximately 0.2 g, were cut into pieces and weighed with an accuracy of ±0.5 mg, and designated W_0_. They were covered with filter paper, placed on a nickel mesh, and their total weight was determined as W_1_. These were placed in a triple-neck flask, refluxed with dichloromethane for 48 h with the solvent changed every 24 h, then washed with ethanol and placed in an oven until constant weight, W_2_, was reached. The gel content, G [%], was determined using the following formula:

G = [1− ( W_1_ − W_2_)/W_0_] × 100% (1)

### 2.5. Characterization

Dynamic mechanical analysis (DMA) measurements were performed on a DMA +450 analyzer (01db-Metravib, Paris, France). The PU film was cut into 4 × 1 × 0.2 cm rectangular strips and tested in tensile mode over the temperature range from −40 to 60 °C. The heating rate was fixed at 3 °C/min. The frequencies were and 0.5, 1, 3 and 5 Hz respectively.

FTIR spectra were recorded with a VERTEX 70 spectrometer (Bruker Daltonics Inc., Billerica, MA, USA) in the range of 4000–500 cm^−1^ at a resolution of 4 cm^−1^; eight scans were collected per sample.

TEM images were recorded with a Tecnai G2 transmission electron microscope with 200 kV accelerating voltage (FEI Co., Hillsboro, OR, USA). Specimens with a thickness of ca. 50 nm were prepared by ultra-cryomicrotomy at −35 °C using a Leica UCT microtome.

SEM and EDS images were taken using a Philips XL-30 FEG (Amsterdam, The Netherlands). The samples were broken in liquid nitrogen, and all samples were coated with an ultrathin Au film by high-vacuum evaporation before observing the cross sections. At least three sections were observed to show a representative image.

Tensile strength was determined by universal testing machine INSTRON 1121 (INSTREAM Corporation, Boston, MA, USA), according to GB/T1040.1-2006. The samples (dumbbell shape, 50 × 2 mm, gauge length 4 mm) were tested at a strain rate of 100 mm/min.

Gel permeation chromatography (GPC) analyses were carried out using PL-GPC-120 from Polymer Laboratories (Lanarkshire, UK). The solvent used was DMF at 80 °C with a flow rate of 1.0 mL/min.

^1^H-NMR and ^13^C-NMR analyses were conducted on a BRUKER 400 MHz NMR spectrometer (Bruker Daltonics Inc.) in CDCl_3_ solvent with tetramethylsilane as the internal standard.

## 3. Results

### 3.1. Chemical Structural Analysis of POSS–PECH

At room temperature, T_7_-POSS transformed from the solid to liquid state after polymerization (see Figure 2a,b). The FTIR spectrum of POSS–PECH presented in Figure 2 shows a peak at approximately 3442 cm^−1^ that was assigned to the O–H stretching band. The peaks at 2958 and 2876 cm^−1^ represent a C–H stretching band. The characteristic peaks at approximately 1430, 1130 and 747 cm^−1^ correspond to deforming of the C–H, C–O–C stretching band and C–Cl stretching band, respectively. The peak at 1596 cm^−1^ is characteristic of phenyl on POSS substituents.

NMR spectra were useful for determining the material composition. Figure 2 presents ^1^H-NMR (2d) and ^13^C-NMR(2e) spectra of POSS–PECH. The peaks at 3.62, 3.72 and 3.99 ppm in Figure 2d correspond to the main chain hydrogen atom of POSS–PECH. The areas of signals at 7.10–7.95 ppm can be related to phenyl on POSS substituents. The ^13^C-NMR spectrum of POSS–PECH is shown in Figure 2e. The assignment of individual peaks is as follows: δ(CH_2_–Cl) is at 43.13–45.07 ppm, δ(O–CH_2_) is at 68.98–71.13 ppm, δ(O–CH) is at 78.60–78.98 ppm, and δ(phenyl)is at 127.53–133.92 ppm.

The above analyses of FTIR and NMR spectra prove that the synthesized product was an oligomer of hydroxyl-terminated polyepichlorohydrin grafted to the POSS structure. The molecular weights of alternative POSS–PECH samples that were synthesized are shown in Table 3. The hydroxyl value of POSS–PECH, as seen in Table 4 and measured by the acetic anhydride–pyridine method, decreases with increasing molecular weight.

### 3.2. Structure and Phase Morphology Analysis of POSS-Based Polyurethane Nanocomposites

**Figure 3:**
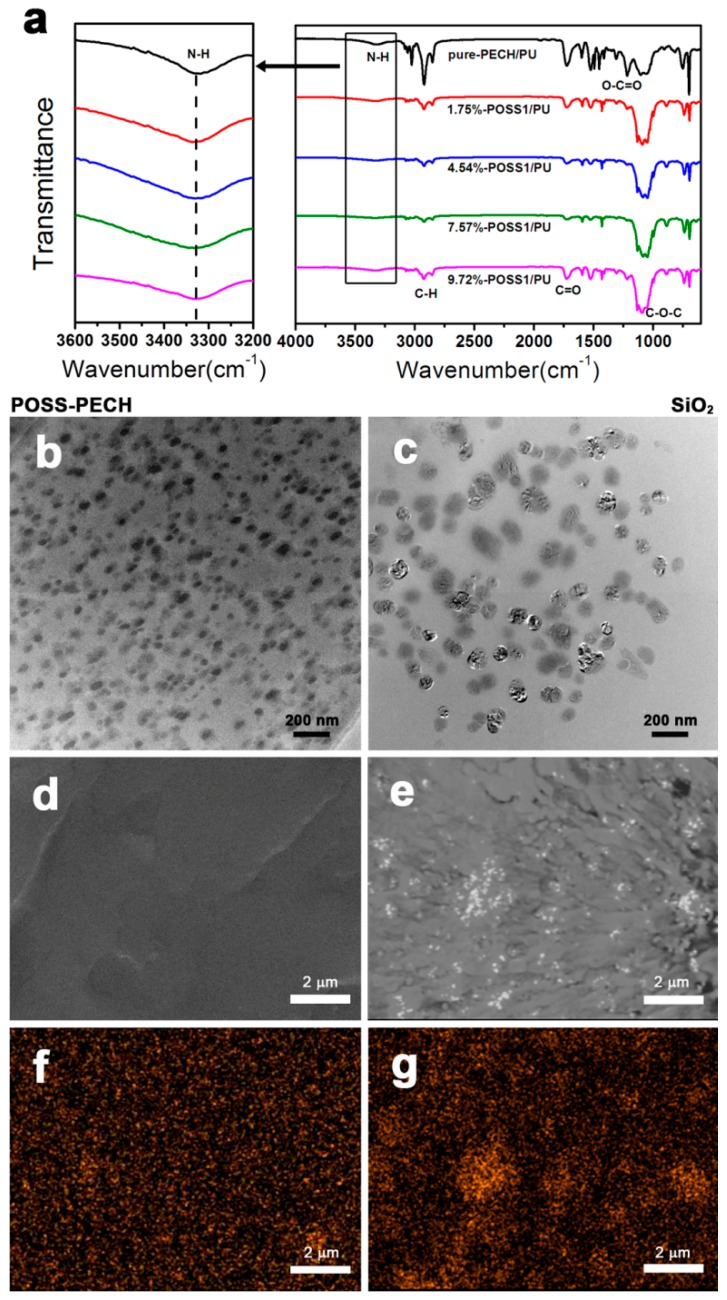
(**a**) FTIR spectra of PUs with different POSS contents. (**b**) TEM images of 9.72% POSS-modified PU. (**c**) TEM images of 9.74% SiO_2_-modified PU. (**d**) SEM images of 9.72% POSS-modified PU. (**e**) SEM images of 1.96% SiO_2_-modified PU. (**f**) Element mapping images of Si 9.72% POSS-modified PU. (**g**) Element mapping images of Si 1.96% SiO_2_-modified PU.

To investigate the chemical structure of the PUs with respect to various contents of POSS–PECH and pure-PECH, their FTIR spectra were obtained and are shown in Figure 3a. The absorption peak of the –NH bond appears at 3329 cm^−1^, and the stretching vibration peak of the C=O bond in the urethane bond appears at 1727 cm^−1^. The characteristic absorption peak of the ether bond C–O–C appears at approximately 1112 cm^−1^. These three features indicate that the characteristic functional group of polyurethane carbamate, NHCOO, was successfully formed. The absence of an absorption peak from isocyanate groups at 2270 cm^−1^ reflects the complete curing reaction.

The dispersion of POSS nanoparticles in the PU composites was observed by TEM. The results show that even when the POSS content reached 9.72 wt % of the total weight of the composites, no POSS particles were agglomerated, and all were uniformly distributed in the composite resin with a nearly identical size. The fracture surface of the composite presented in Figure 3d demonstrates a homogeneous morphology with no phase separation observed between the POSS particles and matrix resin. The results of EDS (see Figure 3f) are in good agreement with those of TEM; that is, the silicon elements in the POSS cage are uniformly distributed throughout the cross section of the sample, with no obvious agglomeration observed.

The above results are substantially different from the traditional morphology and structure of nano-SiO_2_–PU composites. Figure 3c shows a typical TEM micrograph of nanocomposites comprising nanosilica in the PU matrix. The results demonstrate that even if the content of nano-SiO_2_ is only 1.96 wt % of the total composite weight, agglomeration always occurs, and the cluster size after agglomeration is between 40 and 150 nm. It is also observed that the particle dispersion within clusters is not uniform. Consequently, the morphology of fracture surfaces in silica–PU composites (Figure 3e) is notably different from those containing modified POSS. The sharp interface between agglomerated SiO_2_ particles and matrix resin is clearly visible, with phase separation consistent with the agglomeration and non-uniform dispersion of elemental silicon clearly shown in EDS in Figure 3g.

The above results show that the PECH oligomeric chain grafted onto the POSS cage effectively changed the particle surface structure, overcoming the agglomeration problem of POSS particles and facilitating uniform dispersion of POSS inorganic cages within the polyurethane polymer in a monodispersed state [26,27].

### 3.3. Damping Properties

The dynamic properties of materials, including their damping performance, are commonly studied by DMA, in which the storage modulus (E’) and the loss modulus (E”) of the sample under an oscillating load are monitored against time, temperature and frequency of the oscillation. These moduli change with frequency and temperature as the molecular motions within the polymer change. The ratio of E’/E” = tan *δ*, which defines the inherent energy dissipation ability of the material, is commonly used to characterize its damping ability. A high tan *δ* value and large temperature range indicates excellent damping performance of the system.

In general, the energy dissipation reaches a maximum near the glass transition temperature of the polymer (*T*_g_) because in this regime, without considering the chemical structure of the molecular chain, the following molecular chain motions simultaneously transform mechanical energy into thermal energy. When movable molecular chains are relatively short, under the action of external strain, there will be a large conformational change leading to the conformational entropy of materials. In the glassy state, the distance between chains is short and the interaction between chains is strong. Therefore, when the free chains move, the interaction force between the molecules that needs to be overcome is high. When the free chains move relative to each other, the internal friction between the molecular chains that needs to be overcome is large, and the internal friction between the molecular chain and the filler is also large.

#### 3.3.1. Effect of POSS1 Content on the Damping Properties of PU Composites

**Figure 4:**
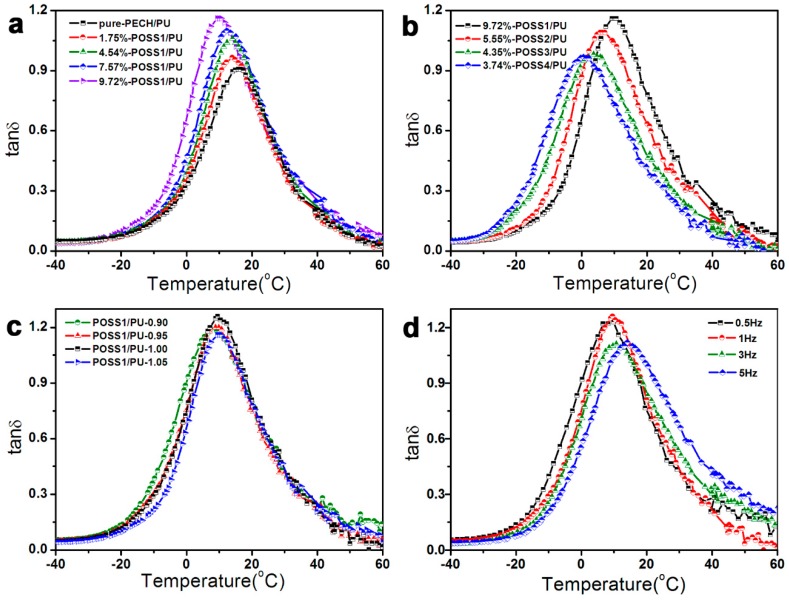
This loss factor (tan *δ*) curves vs temperature for: (**a**) The neat PU matrix and PUs with different POSS contents (POSS–PECH with same molecular weight), (**b**) PUs with different POSS contents (POSS–PECH with different molecular weights), (**c**) PUs with different isocyanate indexes (R) and (**d**) PUs response to different strain frequencies.

The effect of POSS1 content on tan *δ* of PU nanocomposites is shown in Figure 4a and Table 5. The transition peaks in the DMA spectra of PU nanocomposites with different POSS1 contents indicate that POSS–PECH has good compatibility with the matrix material. Compared with pure polyurethane polymer, the tan *δ* of POSS-based PU nanocomposites increased from 0.91 to 1.16 with the increasing POSS content (an increase of 27.5%), whereas the damping temperature range (tan *δ* > 0.3) increased from Δ*T* = 34.4 to 44.0 °C, thus increasing by 28.1%. Meanwhile, the *T*_g_ and the initial temperature of the damping temperature range (*T*_1_) of the PU–POSS nanocomposites gradually decreased respectively: *T*_g_ to 9.4 °C from 15.8 °C and *T*_1_ to –6.9 °C from –1.1 °C. These results demonstrate that monodispersed nano-POSS particles effectively improved the damping properties of the composites, importantly demonstrating that the increased POSS content did not restrict the thermal mobility of molecular chains.

#### 3.3.2. Effect of Molecular Weight Change of POSS–PECH on Damping Property of Composites

The effects of POSS–PECH polyols with different molecular weights on the damping properties of nanocomposites were investigated, as shown in Figure 4b and Table 6. The results demonstrate that the tan *δ* and Δ*T* of all POSS–PECH composites were higher than those of the unmodified bulk material. The tan *δ* decreased gradually with increasing molecular weight of POSS–PECH; although the Δ*T* was almost unchanged, the *T*_1_ of the damping temperature range decreased gradually with increasing molecular weight.

At the same time, the *T*_g_ of composites gradually decreased with increasing molecular weight of POSS–PECH. The initial *T*_g_ was 15.8 °C for pure PU–PECH and decreased to 0.6 °C for composites with POSS4 modified with PECH and a M_w_ = 4136; this is the highest range for the materials investigated in this work. This trend highlights the fact that the size of the interfacial region surrounding the POSS–PECH nanoparticles appears to be a key factor controlling the viscous properties of the system and thus the composite damping properties.

#### 3.3.3. Effect of R Value on Damping Property of Polyurethane Polymer

The R value is the molar ratio of isocyanate groups (–NCO) to hydroxyl groups (–OH). By adjusting the R value, the crosslinking density and structure of composites can be custom-tailored, and hence, it was anticipated that the damping properties could also be adjusted. The numerical data in Table 7 and those graphically presented in Figure 4c provide insight into the damping properties of POSS-based polyurethane composites with different R-values and almost constant contents of POSS nanoparticles. The results demonstrate that the damping properties of POSS-based PU nanocomposites, that is, the tan δ and Δ*T* were almost independent of the R value. Only at R = 1.00 was the loss factor tan *δ* = 1.26, thus showing some improvement of damping properties. However, with the increase in the R value, the Δ*T* was shifted towards higher temperatures, that is, from (−12.3 ↔ +35.8 °C) to (−6.9 °C ↔ +37.1 °C). This is because the content of the hard segment increased slightly with the increase in R value, resulting in the Δ*T* moving towards a high temperature. These results also indicate that the improvement of the damping properties of POSS–PECH based nanocomposites is mainly due to the change in PECH-grafted POSS particle content.

#### 3.3.4. Effect of Different Testing Frequencies on Damping Property of Polyurethane Polymer

The effects of different test frequencies on the damping properties of polyurethane polymers were analyzed using data sets presented in Figure 4d and Table 8. The results show that with increasing test frequency, the tan *δ* of the polymer first increased and then decreased. The *T*_g_ gradually increased, the Δ*T* moved towards high temperature, and the range of tan *δ* > 0.3 increased slightly.

## 4. Discussion

**Figure 5:**
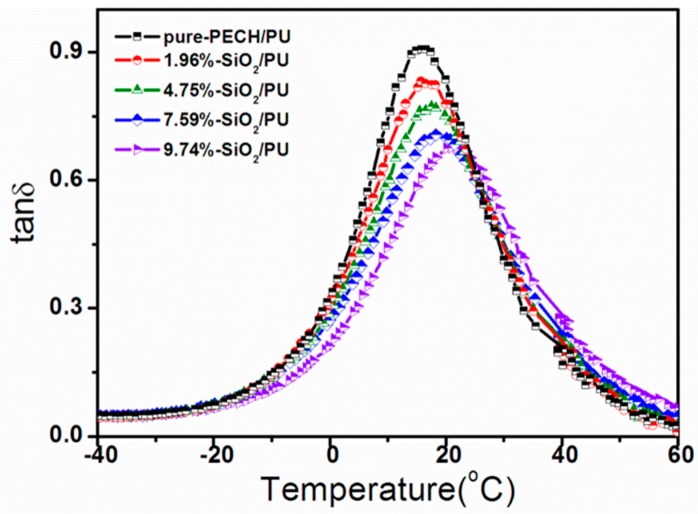
Loss factor (tan *δ*) curves vs temperature for PU composites with the addition of traditional nano-SiO_2_ (0 to 9.74%)

The analysis of the results presented above yields the following information:

When the POSS nanoparticles surface-grafted with pendent chains of PECH are distributed in the polymer matrix in the form of a monodispersed phase, the damping properties of the nanocomposites increase with the increase the nano-POSS content. This effect is completely opposite to the performance of traditional nanocomposites, such as nano-SiO_2_–PU and unmodified POSS–PU composites (for details see Supplementary Materials), whose damping properties are presented in Table 9 and depicted in Figure 5. These results clearly demonstrate that increasing the nano-SiO_2_ content results in the deterioration of the damping properties of SiO_2_–PU nanocomposites. The tan δ is shown to be gradually reduced by 25.3% from the initial value of tan δ equal to 0.91 for the unmodified (pure PECH–PU) material to tan δ equal to 0.68 exhibited by the nano-SiO_2_–PU composite containing 9.74% of SiO_2_ nanoparticles. This loss function value equals only 50% of the damping capability of our composites, with POSS1–PU containing a similar concentration of surface-grafted POSS nanoparticles. The Δ*T* is almost unchanged compared with that related to the original material, but the *T*_1_ and the *T*_g_ of the SiO_2_-composite system both increase with the increasing amount of silica.

A number of earlier studies [28,29] have shown that the decrease in damping performance of traditional SiO_2_–PU composite systems is due to nanoparticles being dispersed in the soft matrix and acting as ‘physical crosslinks’, which limit the thermal movements of molecular chains in the soft matrix. These restrictions become stronger as the number of nanoparticles increases, and hence, the number of ‘physical crosslinks’ is increased. Consequently, any restrictions on the molecular thermal motion ability will lead to a decrease in the damping property of the composite material and an increase in *T*_1_ and *T*_g_.

### 4.1. Analysis of the Mechanism(s) of T_g_ and T_1_ Changes in POSS-Based Polyurethane Nanocomposites

For the amorphous POSS–PU nanocomposite systems, the change of *T*_1_ and *T*_g_ is closely related to the crosslinking density, molecular weight between crosslinking points and the interaction between molecular chains. For this reason, we determined the following important properties of our materials: (i) The crosslinking density of the systems, which was tested by gel extraction (see Section 2.4 for details), and (ii) the change of cohesive energy density (CED) of POSS–PU and SiO_2_–PU composite systems, which is used to estimate the intermolecular interaction in polymers and can be estimated by formula (2) [30,31]:

E = 13.3 δ^2^, (2)

where E is the modulus of elasticity (as determined through mechanical testing of our composites); see data in Table 10 and Table 11.

The analysis of data in Table 10 and Table 11 highlights the following:

The gel content of the PU composite decreased with the increase of POSS1 content. This indicated that the chemical crosslinking degree of the system was also decreasing. However, SiO_2_ with similar content had little influence on the gel content of the system. Therefore, compared with SiO_2_–PU composites, the molecular chain thermal motions of the POSS–PU system increased with the increase of increasing POSS content due to the limitation of the decreased limitations of chemical and physical crosslinking decrease.

The CED of the PU composites decreased with increasing POSS1 content. The CED of the POSS–PU system decreased from 820 KPa (for an unmodified PU) to 400 KPa (9.72 wt % POSS1). However, the CED (a measure of the strength of molecular chain interactions) of the SiO_2_ composite system with similar content increased to 2400 KPa. The decreasing CED indicated that in the monodispersed state, even if the POSS particles content reached 9.72 wt %, it will not hinder the thermal motion of PU molecular chains.

The influence of gel content and CED on the thermal motion ability of the molecular chains of the POSS–PU system was consistent. That is, with the increase of POSS content, chemical and physical restrictions in the system were constantly reduced. Hence, the thermal motion ability of the PU molecular chain segment was enhanced. As a result, *T*_1_ and *T*_g_ decreased with the increasing POSS content.

### 4.2. Analysis of the Damping Mechanism of the POSS-Based Polyurethane Composite System

**Figure 6:**
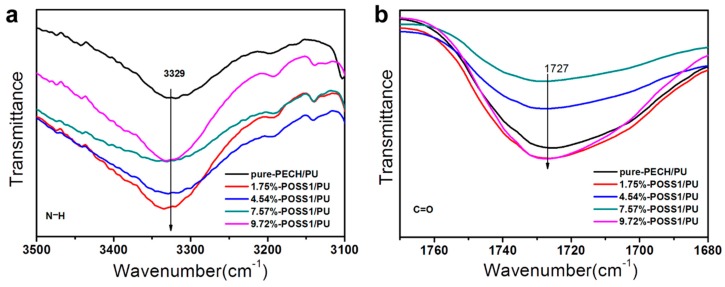
FTIR spectra of the N–H stretching regions and the C=O stretching regions of PUs with different contents of POSS–PECH.

The damping properties of polymeric materials arise from the internal friction caused by molecular chain motions and the formation and dissociation of intermolecular hydrogen bonds. The structural chemical characteristics of our nanocomposite systems were determined through FTIR, which identifies the hydrogen bonds in the analyzed systems. For the PUs, hydrogen bonding appeared in the N–H stretching region (3500–3100 cm^−1^) and the C=O stretching region (1740–1710 cm^−1^), in which two absorption peaks are observed. The spectra of the stretching vibrations of N–H and –C=O in polymers with different POSS–PECH contents at the same molecular weight of POSS–PECH are shown in Figure 6. As shown in Figure 6, the stretching vibration peaks of weakly hydrogen bonded N–H and non-hydrogen bonded –C=O appeared at 3329 cm^−1^ and 1727 cm^−1^, respectively, and the peak position did not shift with the change of POSS–PECH content.

In other words, the increase in the POSS core contents did not cause a change in the hydrogen bonding between the molecular chains. Therefore, the damping factor of the POSS–PU composite material was mainly caused by the friction between the PU molecular chain and the inorganic nano-POSS cage surface.

The fraction of interfacial area between our PECH-functionalized hybrid nanoparticles and the polymer matrix increased with increasing such POSS content. Under the action of periodic external forces, the total amount of PU molecular chains in the interfacial region that were repeatedly stripped and re-adsorbed also kept increasing, which results in the damping factor increasing with the increase of our PECH–POSS content.

## 5. Conclusions

Although polymers often exhibit good damping ability near the *T*_g_, they generally exhibit a relatively low (tan *δ*) and narrow Δ*T* because they lack extreme and continuous composition heterogeneity and thus cannot serve as damping materials in a broad temperature range. Considering the above issues, in this work we accomplished alleviating the above problems through: (i) Preparation of homogeneous monodispersed polymer nanocomposites utilizing bio-based PU resin as the matrix and (ii) significant broadening of the damping temperature range. 

We demonstrated here that POSS surface-grafting with oligomeric chains of variable molecular weight yields liquefied hydroxyl-terminated polyepichlorohydrin with a POSS structure. The new organic–inorganic hybrid material enables facile preparation of mono-dispersed nanocomposite systems achieving (i) total dispersion of nanoparticles providing excellent polymer nanocomposite homogeneity for POSS contents in the range 1.75 to 9.75%, (ii) excellent damping properties achieved through improvement of viscoelastic properties of interfacial zone achieved through shielding of inorganic (POSS) core by surface-grafted oligomeric molecular chains, and (iii) significant reduction of *T*_g_, combined with broadening of damping temperature field. The proposed approach lays the foundation for a novel means of engineering the glass transition breadth and damping properties of elastomeric nanocomposites over a broadened range of application temperatures.

## Figures and Tables

**Table 1:** Feed molar ratio of POSS–PECH with different molecular weights.

PECH Samples	T_7_-POSS (mol)	ECH (mol)	BF_3_·OEt_2_ (mol)	Mw of POSS–PECH
POSS-1	1	10	0.6	1573
POSS-2	1	25	0.6	2762
POSS-3	1	40	0.6	3533
POSS-4	1	55	0.6	4136

**Table 2:** Formulations of PU compositions

PU Samples	Castor Oil (g)	PECH (g)	POSS1 (g)	POSS2 (g)	POSS3 (g)	POSS4 (g)	PAPI (g)
Pure-PECH/PU	10	10					6.09
1.75%-POSS1/PU	10	10	2				6.28
4.54%-POSS1/PU	10	10	6				6.66
7.57%-POSS1/PU	10	10	12				7.22
9.72%-POSS1/PU	10	10	18				7.79
5.55%-POSS2/PU	10	10		18			7.68
4.35%-POSS3/PU	10	10			18		7.60
3.74%-POSS4/PU	10	10				18	7.48
POSS1/PU-0.90	10	10	18				6.67
POSS1/PU-0.95	10	10	18				7.05
POSS1/PU-1.00	10	10	18				7.42
POSS1/PU-1.05	10	10	18				7.79

**Table 3:** Summary of gel permeation chromatography (GPC) data of POSS–PECH.

PECH Samples	Mp	Mn	Mv	Mw	Mz	Mz + 1	PD
POSS-1	1607	1348	1538	1573	1812	2057	1.1669
POSS-2	2120	1960	2628	2762	3737	4698	1.4092
POSS-3	3841	2439	3365	3533	4689	5730	1.4485
POSS-4	4871	2614	3894	4136	5849	7420	1.5822

**Table 4:** Hydroxyl value of POSS–PECH with different molecular weights.

PECH Samples	Hydroxyl Value (mol/g）
POSS-1	6.64 × 10^−4^
POSS-2	6.20 × 10^−4^
POSS-3	5.90 × 10^−4^
POSS-4	5.42 × 10^−4^

**Table 5:** Summary of DMA data of PUs with different POSS contents (POSS–PECH with same molecular weight).

PU Samples	Inorganic Core Content (%)	tan *δ T*_g_/°C		Damping Temperature Field (tan δ > 0.3)		
				*T*_1_/°C	*T*_2_/°C	Δ*T*°C
Pure-PECH/PU	0	0.9064	15.8	-1.1	33.3	34.4
1.75%-POSS1/PU	1.75	0.9659	14.0	-2.0	32.6	34.6
4.54%-POSS1/PU	4.54	1.058	14.1	-2.7	34.2	36.9
7.57%-POSS1/PU	7.57	1.099	12.3	-4.0	37.4	41.4
9.72%-POSS1/PU	9.72	1.164	9.4	-6.9	37.1	44.0

M_w_ = 1573, R = 1.05, the percentage content is the content of inorganic POSS nanoparticles. *T*_1_ is the starting temperature of tan *δ* > 0.3, and *T*_2_ is the terminating temperature of tan *δ* > 0.3.

**Table 6:** Summary of DMA data of PUs with different molecular weights of POSS–PECH.

PU Samples	Inorganic Core Content (%)	tan *δ T*_g_/°C		Damping Temperature Field (tan *δ* > 0.3)		
				*T*_1_/°C	*T*_2_/°C	Δ*T*°C
Pure PECH/PU 9.72%-POSS1/PU	0 9.72	0.964 1.164	15.8 9.4	−1.1 −6.9	33.3 37.1	34.4 44.0
5.55%-POSS2/PU	5.55	1.091	6.5	−10.3	34.0	44.3
4.35%-POSS3/PU	4.35	0.9813	3.7	−15.1	28.2	43.3
3.74%-POSS4/PU	3.74	0.9681	0.6	−18.1	25.9	44.0

*T*_1_ is the starting temperature of tan *δ* > 0.3, and *T*_2_ is the terminating temperature of tan *δ* > 0.3.

**Table 7:** Summary of DMA data of PUs with different molecular weights of POSS–PECH.

PU Samples [POSS/PU–R]	Inorganic Core Content (%)	tan *δ T*_g_/°C		Damping Temperature Field (tan *δ* > 0.3)		
				*T*_1_/°C	*T*_2_/°C	Δ*T*°C
POSS1/PU-0.90	9.96	1.186	7.5	−12.3	35.8	48.1
POSS1/PU-0.95	9.88	1.201	9.6	−10.4	33.6	44.0
POSS1/PU-1.00	9.80	1.262	9.6	−9.7	35.1	44.8
POSS1/PU-1.05	9.72	1.164	9.4	−6.9	37.1	44.0

*T*_1_ is the starting temperature of tan *δ* > 0.3 and *T*_2_ is the terminating temperature of tan *δ* > 0.3.

**Table 8:** Summary of DMA data of PUs with different test frequencies.

Test Frequency (Hz)	Tan *δ T*_g_/°C		Damping Temperature Field (tan *δ* > 0.3)		
			*T*_1_/°C	*T*_2_/°C	Δ*T*°C
0.5	1.234	9.9	−12.3	34.5	46.8
1	1.262	9.6	−9.7	35.1	44.8
3	1.111	10.7	−8.7	40.1	48.8
5	1.119	14.1	−6.8	48.5	55.3

M_w_ = 1573, R = 1.05, PU with 9.72% inorganic nanomaterials content was tested by DMA at different frequencies.

**Table 9:** Summary of DMA data of PU Composites with traditional nano- SiO_2_.

PU Samples	tan *δ T*_g_/°C		Damping Temperature Field (tan *δ* > 0.3)		
			*T*_1_/°C	*T*_2_/°C	Δ*T*°C
pure-PECH/PU	0.9064	15.75	−1.1	33.3	34.4
1.96%-SiO_2_/PU	0.8319	15.70	−0.8	34.8	35.6
4.75%-SiO_2_/PU	0.7729	17.50	0.2	34.8	34.6
7.59%-SiO_2_/PU	0.7065	18.25	1.2	36.8	35.6
9.74%-SiO_2_/PU	0.6761	21.00	4.4	38.7	34.3

**Table 10:** Mechanical properties and gel content of polyurethane polymers with different POSS–PECH contents.

PU Samples	Tensile Strength (MPa)	Modulus of Elasticity (MPa)	Elongation at Break (%)	Critical Fracture Stress (MPa)	Cohesive Energy Density (MPa)	Gel Content (%)
Pure-PECH/PU	8.32	10.90	142.5	8.32	0.82	96.28
1.75%-POSS1/PU	8.18	9.97	160	8.18	0.75	90.20
4.54%-POSS1/PU	5.70	7.54	140	7.86	0.57	83.18
7.57%-POSS1/PU	4.84	6.26	150	5.21	0.47	78.47
9.72%-POSS1/PU	3.89	5.29	145	3.89	0.40	70.10

**Table 11:** Mechanical properties and gel content of PU composites with different nano-SiO_2_ contents.

PU Samples	Tensile Strength (MPa)	Modulus of Elasticity (MPa)	Elongation at Break (%)	Critical Fracture Stress (MPa)	Cohesive Energy Density (MPa)	Gel Content (%)
Pure-PECH/PU	8.32	10.90	142.5	8.32	0.82	96.28
1.96%-SiO_2_/PU	5.76	12.18	94.0	5.76	0.92	98.24
4.75%-SiO_2_/PU	6.87	17.00	86.7	6.87	1.28	96.67
7.59%-SiO_2_/PU	10.21	23.93	84.7	10.21	1.80	97.39
9.74%-SiO_2_/PU	15.83	31.93	102	15.83	2.40	96.96

## Supplementary Materials

The following are available online at https://www.mdpi.com/2073-4360/11/4/647/s1, Figure S1 ^29^Si NMR spectra of POSS1–PECH Figure S2: FTIR spectra of POSS2–PECH, Figure S3: FTIR spectra of POSS3–PECH, Figure S4: FTIR spectra of POSS4–PECH, Figure S5: ^1^H NMR spectra of POSS2–PECH, Figure S6: ^1^H NMR spectra of POSS3–PECH, Figure S7: ^1^H NMR spectra of POSS4–PECH, Figure S8: ^13^C NMR spectra of POSS2–PECH, Figure S9: ^13^C NMR spectra of POSS3–PECH, Figure S10: ^13^C NMR spectra of POSS4–PECH, Figure S11: ATR-IR spectra of PUs with different POSS–PECH, Figure S12: ATR-IR spectra of PUs with different isocyanate index, Figure S13: ATR-IR spectra of PUs with different SiO_2_ content S14: (**a**) SEM images of 1.75% POSS modified PU. (**b**) Element mapping images of Si (1.75% POSS modified PU). (**c**)SEM images of 4.54% POSS modified PU. (**d**)Element mapping images of Si (4.54%POSS modified PU). (**e**)SEM images of 7.57% POSS modified PU. (**f**)Element mapping images of Si (7.57% POSS modified PU), Figure S15: (**a**) SEM images of 4.75% SiO_2_ modified PU. (**b**) Element mapping images of Si (4.75% SiO_2_ modified PU). (**c**) SEM images of 7.59% SiO_2_ modified PU. (**d**) Element mapping images of Si (7.59% SiO_2_ modified PU). (**e**) SEM images of 9.74%SiO_2_ modified PU. (**f**) Element mapping images of Si (9.74% SiO_2_ modified PU), Figure S16: Loss factor(tan δ) curves vs temperature for PUs with different T_7_-POSS contents, Figure S17: (**a**) SEM images of 1.77% T_7_-POSS modified PU. (**b**) SEM images of 4.52%T_7_-POSS modified PU. (**c**) SEM images of 7.55% T_7_-POSS modified PU. (**d**) SEM images of 9.73% T_7_-POSS modified PU, Figure S18: TEM images of 1.77% T_7_-POSS modified PU, Figure S19: Loss factor(tan δ) curves vs temperature for PUs with different T_8_-POSS contents, Figure S20: (**a**) SEM images of 1.76% T_8_-POSS modified PU. (**b**) SEM images of 4.55% T_8_-POSS modified PU. (**c**) SEM images of 7.57% T_8_-POSS modified PU. (**d**) SEM images of 9.75% T_8_-POSS modified PU, Figure S21: TEM images of 1.76% T_8_-POSS modified PU, Table S1: Formulations of PU compositions, Table S2: Summary of DMA data of PUs with different T_7_-POSS contents, Table S3: Mechanical properties and gel content of PU Composites with different T_7_-POSS content, Table S4: Formulations of PU compositions, Table S5: Summary of DMA data of PUs with different T_8_-POSS contents, Table S6: Mechanical properties and gel content of PU Composites with differentT_7_-POSS content.
