# Supplementary material for: Improving the Damping Properties of Nanocomposites by Monodispersed Hybrid POSS Nanoparticles: Preparation and Mechanisms
Source: Polymers (Basel). 2019 Apr 9;11(4):647. doi: 10.3390/polym11040647 (PMC6523941; doi:10.3390/polym11040647)
Supplement: Supplementary file 1 [file polymers-11-00647-s001.pdf]

Supporting Information

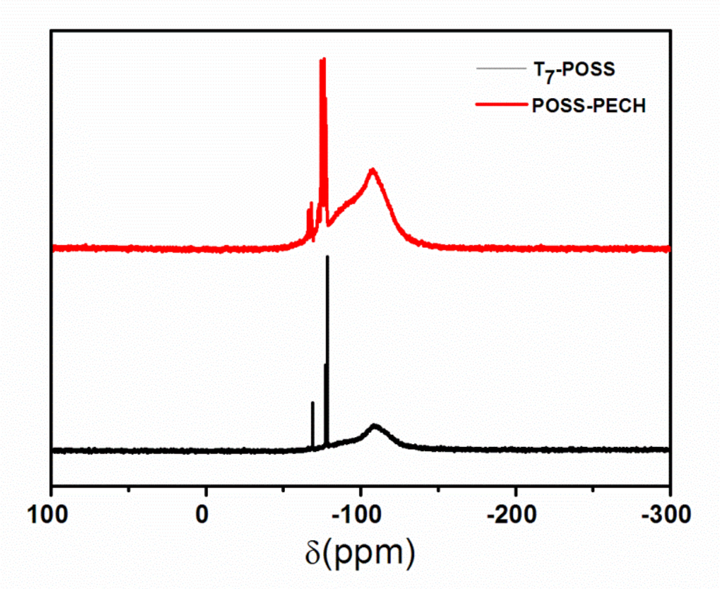

Fig. S1 ^{29}Si NMR spectra of POSS1-PECH

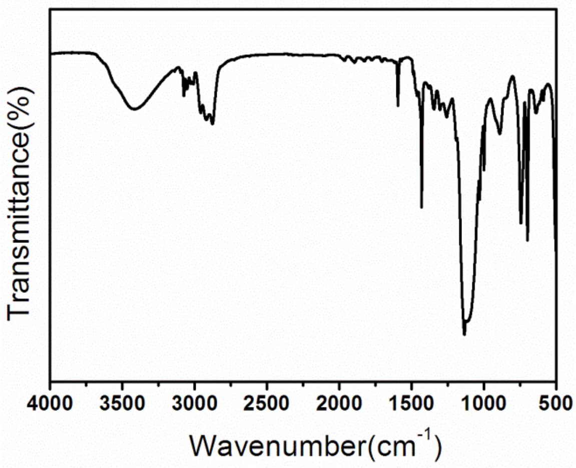

Fig. S2 FTIR spectra of POSS2-PECH

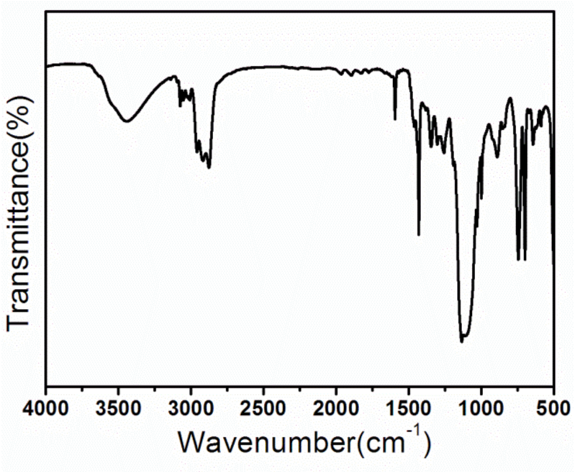

Fig. S3 FTIR spectra of POSS3-PECH

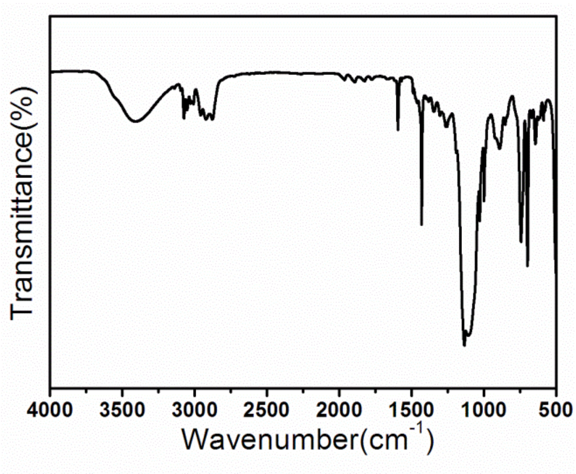

Fig. S4 FTIR spectra of POSS4-PECH

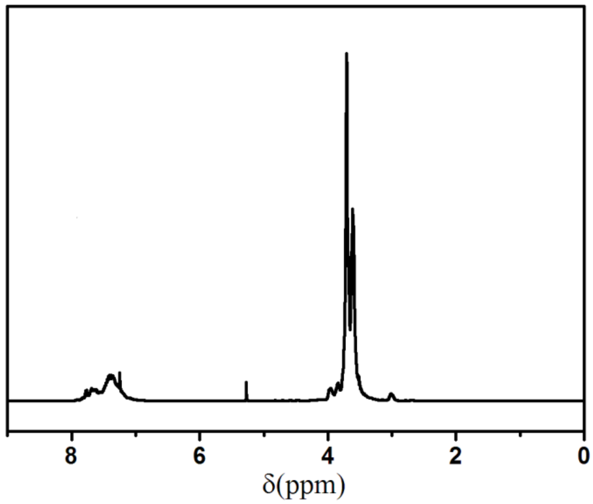

Fig. S5 ^1H NMR spectra of POSS2-PECH

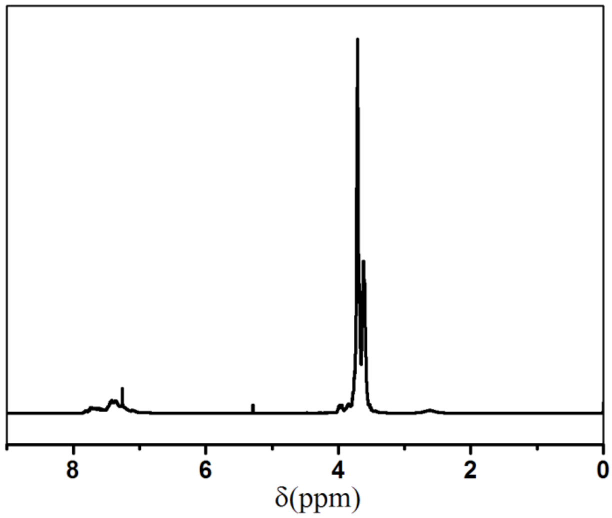

Fig. S6 ^1H NMR spectra of POSS3-PECH

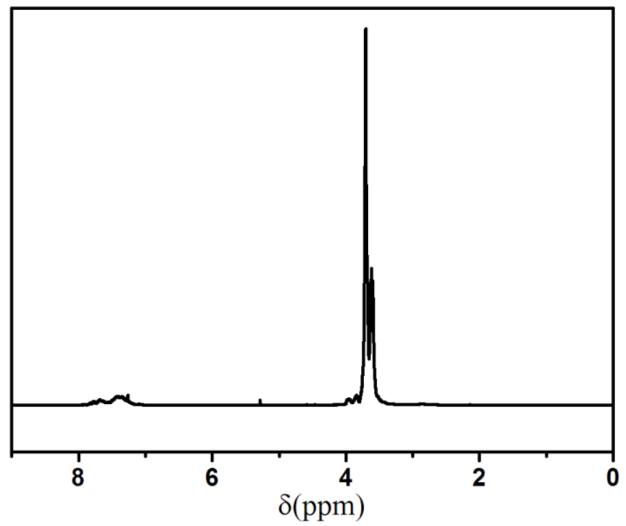

Fig. S7 ¹H NMR spectra of POSS4-PECH

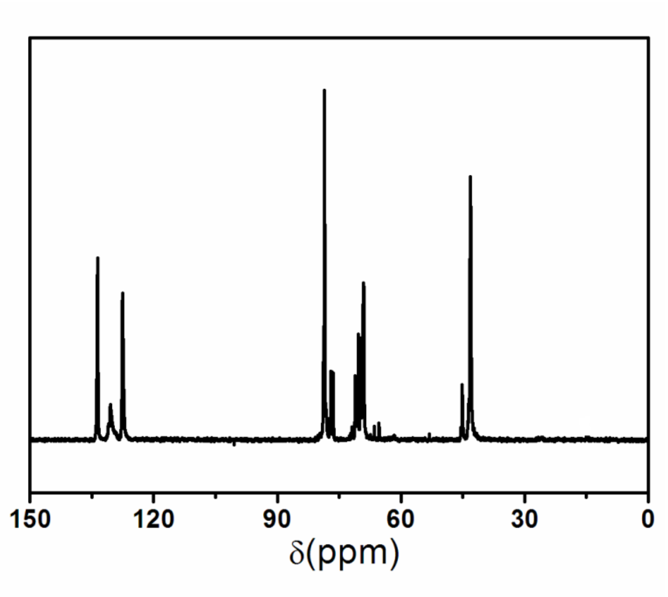

Fig. S8 ¹³C NMR spectra of POSS2-PECH

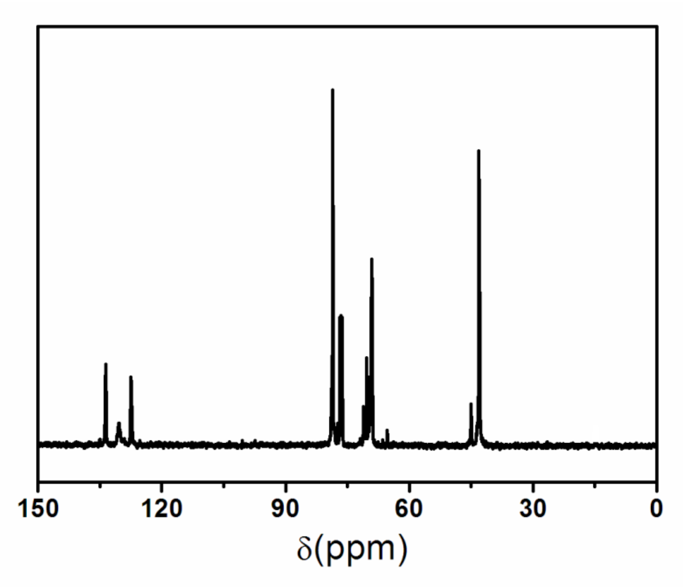

Fig. S9 ^{13}C NMR spectra of POSS3-PECH

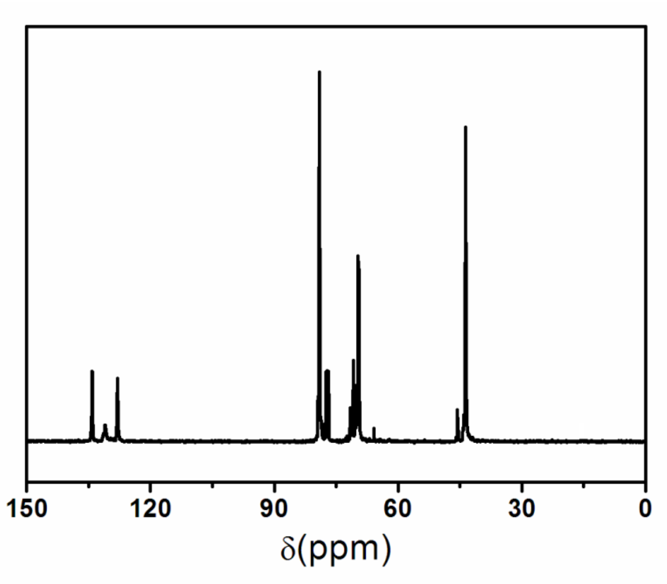

Fig. S10 ^{13}C NMR spectra of POSS4-PECH

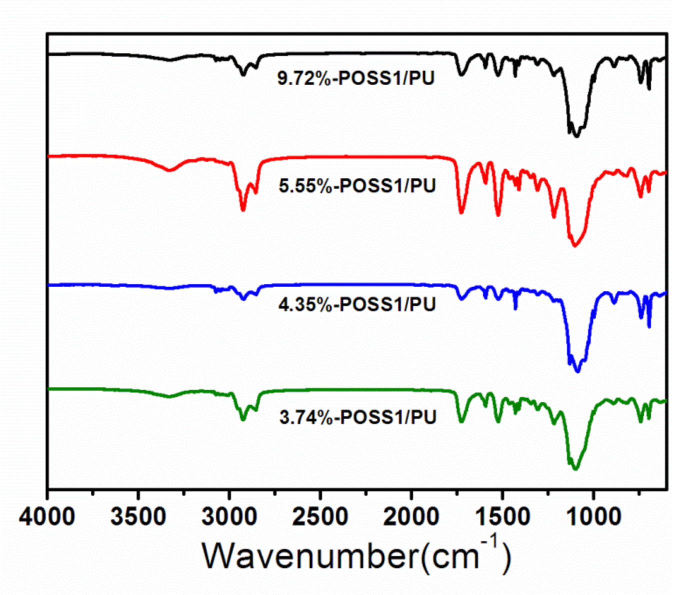

Fig. S11 ATR-IR spectra of PUs with different POSS-PECH

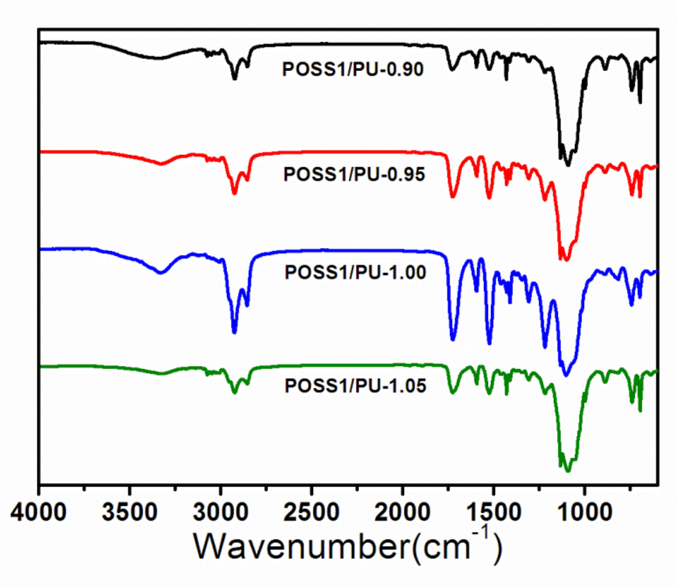

Fig. S12 ATR-IR spectra of PUs with different isocyanate index

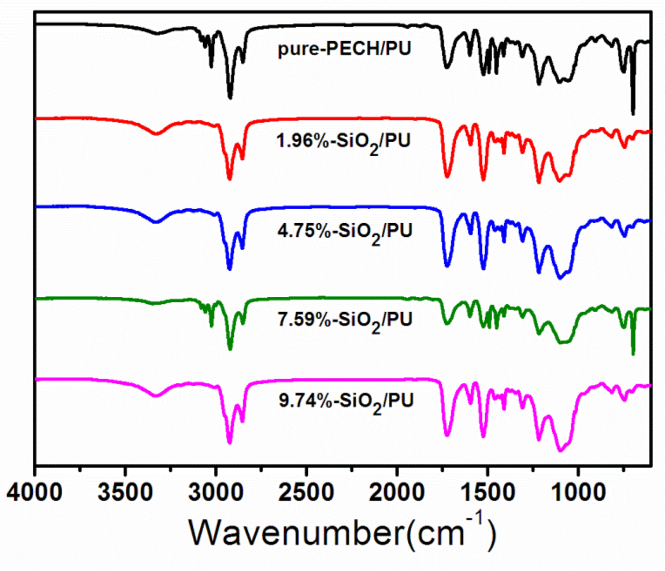

Fig. S13 ATR-IR spectra of PUs with different SiO_2 content

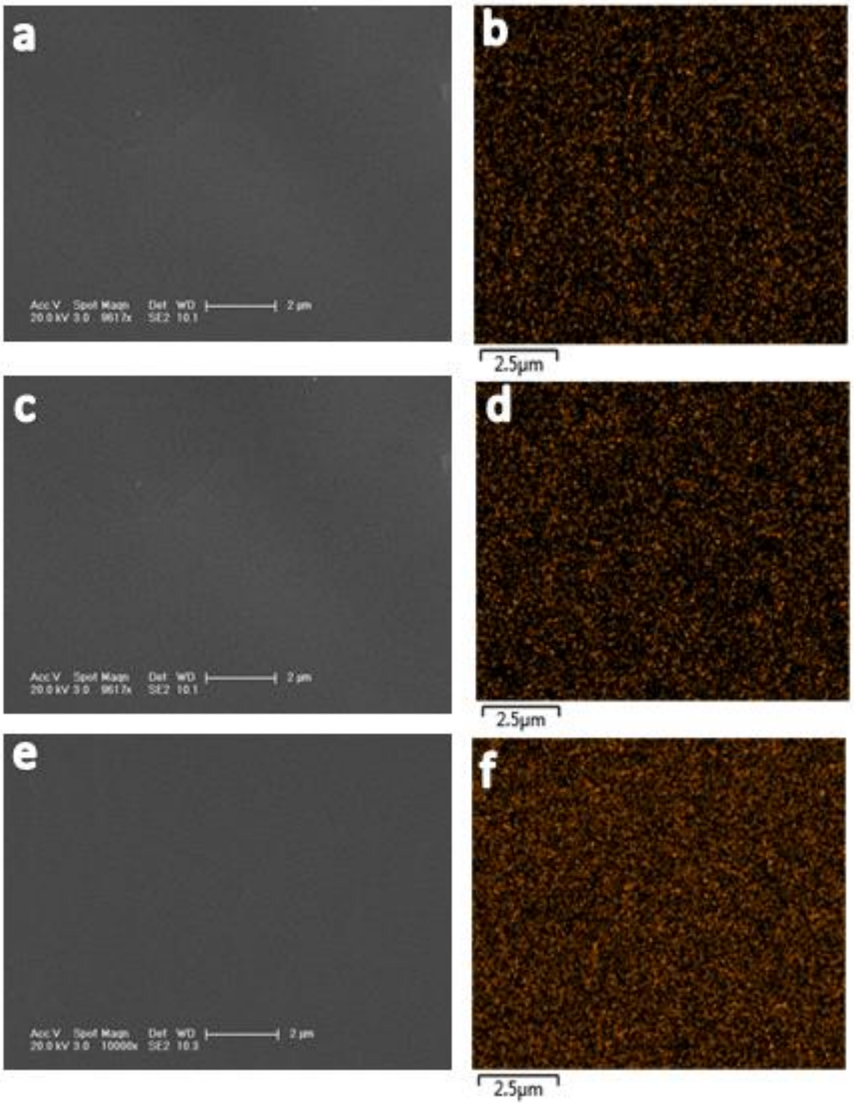

Fig. S14 (a)SEM images of 1.75%POSS modified PU. (b) Element mapping images of Si(1.75%POSS modified PU). (c)SEM images of 4.54%POSS modified PU. (d)Element mapping images of Si(4.54%POSS modified PU). (e)SEM

images of 7.57%POSS modified PU. (f)Element mapping images of Si(7.57%POSS modified PU).

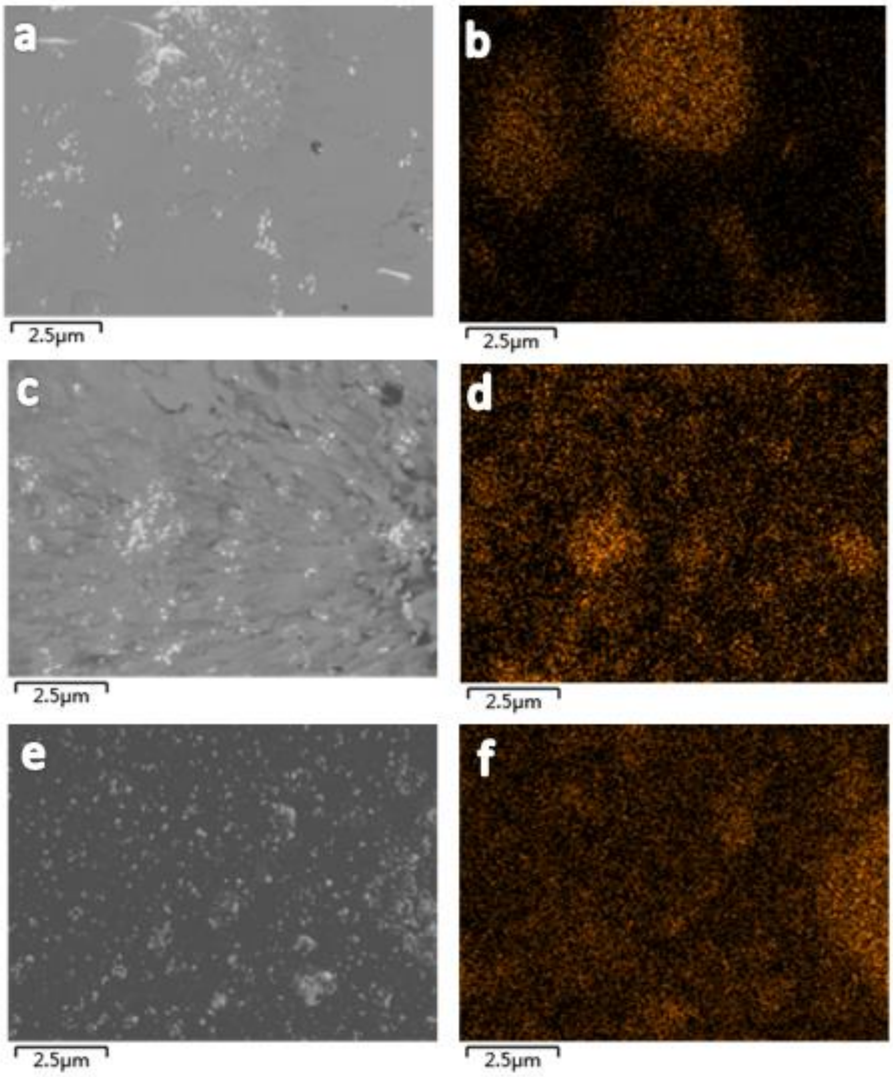

Fig. S15 (a) SEM images of 4.75%SiO_2 modified PU. (b) Element mapping images of Si (4.75%SiO_2 modified PU). (c) SEM images of 7.59%SiO_2 modified PU. (d) Element mapping images of Si (7.59%SiO_2 modified PU). (e) SEM images of 9.74%SiO_2 modified PU. (f)Element mapping images of Si (9.74%SiO_2 modified PU).

Table S1 Formulations of PU compositions

PU samples	Castor oil (g)	PECH(g)	T_7-POSS(g)	PAPI(g)
pure-PECH/PU	10	10		6.09
1.77%-T_7-POSS/PU	10	10	1.18	6.63
4.52%-T_7-POSS/PU	10	10	3.35	7.62
7.55%-T_7-POSS/PU	10	10	6.40	9.02
9.73%-T_7-POSS/PU	10	10	9.20	10.29

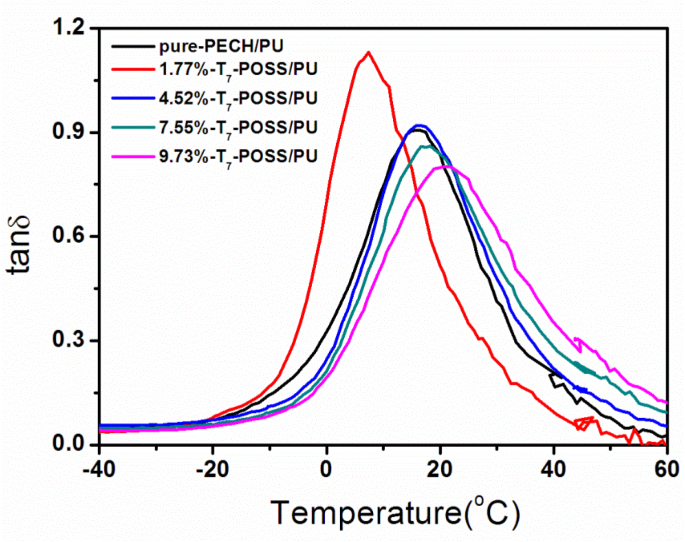

Fig. S16 Loss factor(tanδ) curves vs. temperature for PUs with different T_7-POSS contents

Table S2 Summary of DMA data of PUs with different T_7-POSS contents

PU samples	inorganic core content (%)	tanδ	T_g/℃	Damping temperature field（tanδ > 0.3）		
				T_1/℃	T_2/℃	ΔT/`℃
pure-PECH/PU	0	0.9064	15.75	-1.06	33.25	34.31
1.77%-T_7-POSS/PU	1.77	1.13	7.35	-5.93	27.33	33.26
4.52%-T_7-POSS/PU	4.52	0.9201	16.6	1.37	36.10	34.73
7.55%-T_7-POSS/PU	7.55	0.8572	17.6	2.16	38.84	36.68
9.73%-T_7-POSS/PU	9.73	0.8017	20.75	3.59	42.72	39.13

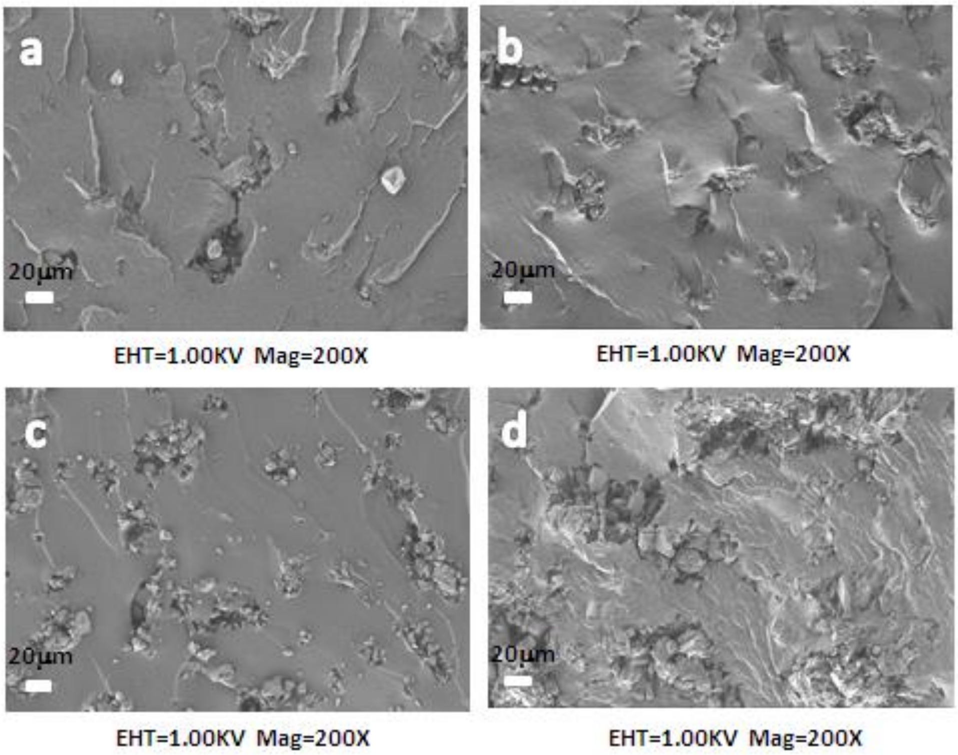

Fig. S17 (a) SEM images of 1.77%T_7-POSS modified PU. (b) SEM images of 4.52%T_7-POSS modified PU. (c) SEM images of 7.55% T_7-POSS modified PU. (d) SEM images of 9.73% T_7-POSS modified PU.

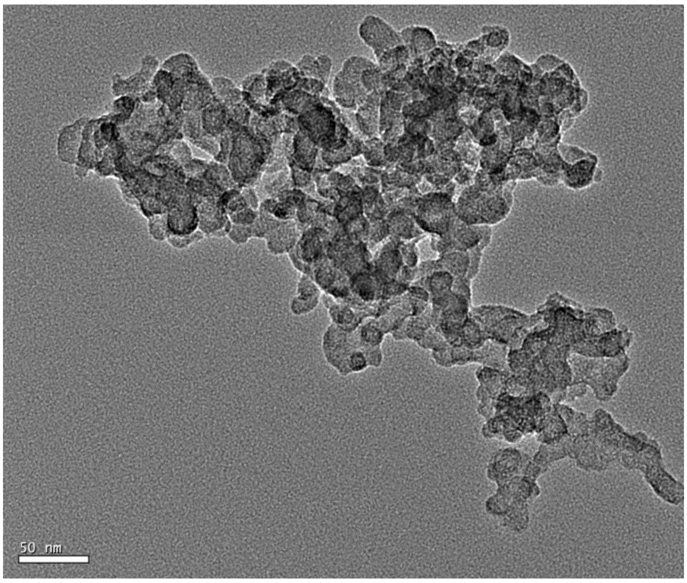

Fig. S18 TEM images of 1.77%T_7-POSS modified PU

Table S3 Mechanical properties and gel content of PU Composites with different T_7-POSS content

PU samples	tensile strength（MPa）	Modulus of elasticity（MPa）	Elongation at break（%）	Critical fracture stress (MPa）	Cohesive energy density（MPa）	Gel content（%）
pure-PECH/PU	8.32	10.90	142.5	8.32	0.82	96.28
1.77%-T_7-POSS/PU	2.22	3.52	120	2.18	0.26	93.30
4.52%-T_7-POSS/PU	2.58	5.32	149	2.55	0.40	85.63
7.55%-T_7-POSS/PU	1.91	6.16	145	1.91	0.46	75.56
9.73%-T_7-POSS/PU	1.50	4.71	169	1.50	0.35	67.55

Table S4 Formulations of PU compositions

PU samples	Castor oil (g)	PECH(g)	T_8-POSS(g)	PAPI(g)
pure-PECH/PU	10	10		6.09
1.76%-T_8-POSS/PU	10	10	1.00	6.09
4.55%-T_8-POSS/PU	10	10	2.77	6.09
7.57%-T_8-POSS/PU	10	10	4.93	6.09
9.75%-T_8-POSS/PU	10	10	6.72	6.09

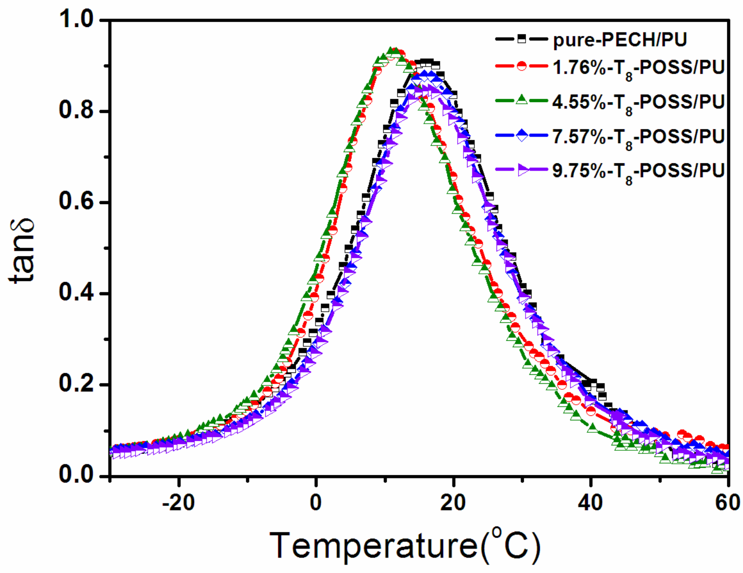

Fig. S19 Loss factor(tanδ) curves vs. temperature forPUs with different T_8-POSS contents

Table S5 Summary of DMA data of PUs with different T_8-POSS contents

PU samples	inorganic core content (%)	tanδ	T_g/℃	Damping temperature field（tanδ > 0.3）		
				T_1/℃	T_2/℃	ΔT/`℃
pure-PECH/PU	0	0.9064	15.75	-1.06	33.25	34.31
1.76%-T_8-POSS/PU	1.76	0.9291	11.5	-2.68	29.88	32.56
4.55%-T_8-POSS/PU	4.55	0.9269	10.75	-3.84	28.73	32.57
7.57%-T_8-POSS/PU	7.57	0.8776	15.70	0.40	33.47	33.07
9.75%-T_8-POSS/PU	9.75	0.8448	15.95	0.36	33.43	33.07

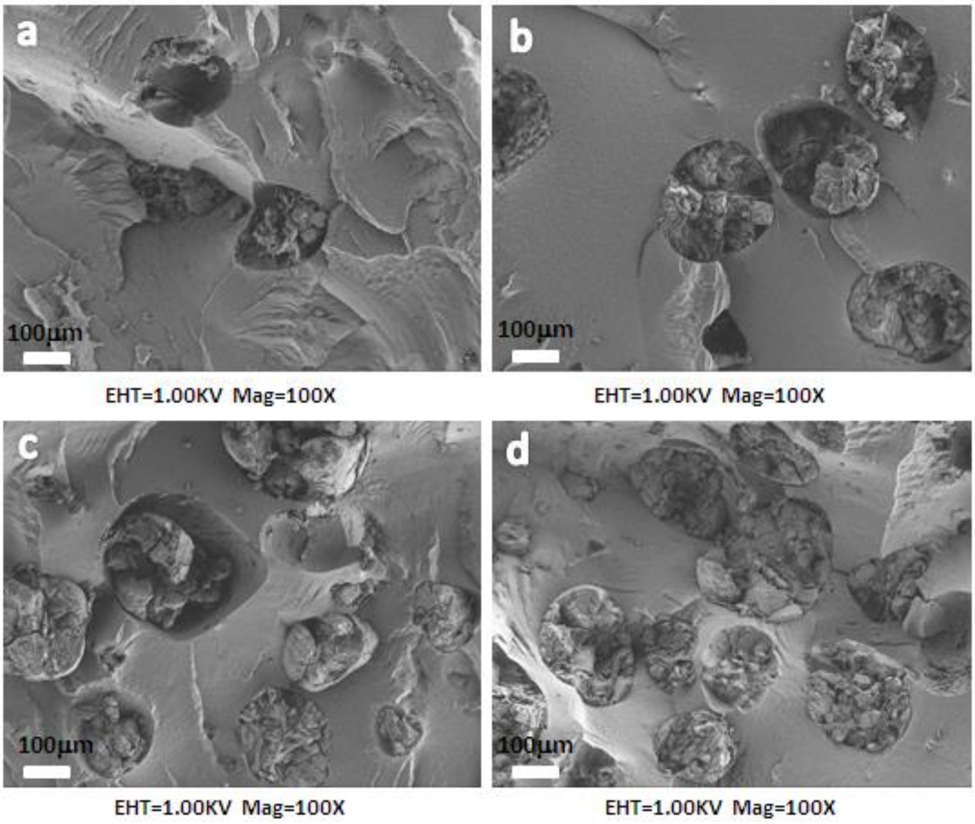

Fig. S20 (a) SEM images of 1.76%T_8-POSS modified PU. (b) SEM images of 4.55%T_8-POSS modified PU. (c) SEM images of 7.57%T_8-POSS modified PU. (d) SEM images of 9.75%T_8-POSS modified PU.

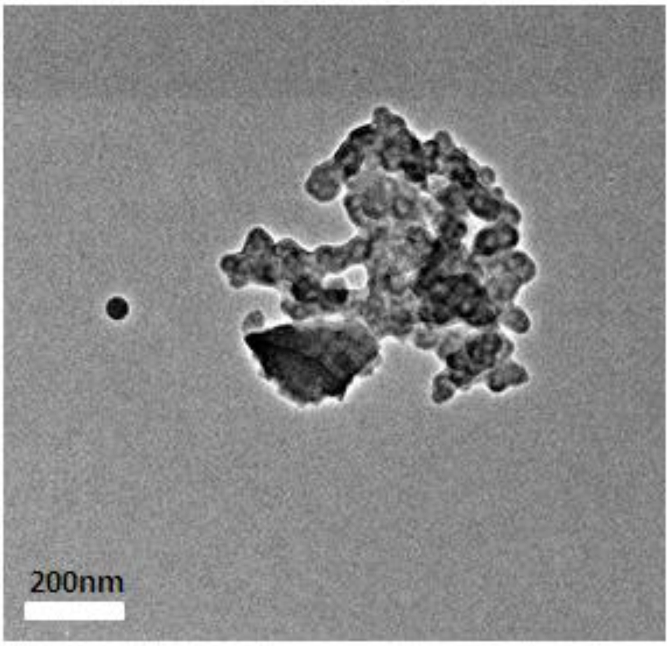

Fig. S21 TEM images of 1.76%T_8-POSS modified PU

Table S6 Mechanical properties and gel content of PU Composites with differentT_7-POSS content

PU samples	tensile strength（MPa）	Modulus of elasticity（MPa）	Elongation at break（%）	Critical fracture stress (MPa）	Cohesive energy density（MPa）	Gel content（%）
pure-PECH/PU	8.32	10.90	142.5	8.32	0.82	96.28
1.76%-T_8-POSS/PU	2.53	4.22	123	2.53	0.32	95.07
4.55%-T_8-POSS/PU	2.09	4.50	107	2.00	0.34	88.36
7.57%-T_8-POSS/PU	1.84	5.28	109	1.74	0.40	82.19
9.75%-T_8-POSS/PU	1.66	5.28	107	1.66	0.40	77.37
